# Transient ischaemic attack: a qualitative study of the long term consequences for patients

**DOI:** 10.1186/s12875-014-0174-9

**Published:** 2014-10-29

**Authors:** Elizabeth J Croot, Tony W Ryan, Jennifer Read, Fiona Campbell, Alicia O’Cathain, Graham Venables

**Affiliations:** Medical Care Research Unit, School of Health and Related Research (ScHARR), University of Sheffield, Regent Court, 30 Regent Street, Sheffield, UK; School of Nursing and Midwifery, University of Sheffield, Barber House Annex, 3a Clarkehouse Road, Sheffield, UK; Health Economics and Decision Science, School of Health and Related Research (ScHARR), University of Sheffield, Regent Court, 30 Regent Street, Sheffield, UK; Neurology Department, Sheffield Teaching Hospitals NHS Foundation Trust, Neurosciences, 12 Claremont Crescent, Sheffield, UK

**Keywords:** Transient ischaemic attack, Qualitative, Patient experience, Self-management, Secondary prevention, Stroke

## Abstract

**Background:**

Transient ischaemic attack (TIA) is characterised by its transient nature with symptoms of neurological dysfunction resolving within 24 hours. The occurrence of TIA is a major risk factor for stroke with 10–15% of TIA patients going on to have ischaemic stroke. Internationally, recommendations for the management of TIA focus on the need for early diagnosis and medical management of the acute increased risk of ischaemic stroke. However there is a limited amount of evidence that some patients suffer enduring consequences as a result of this ‘transient’ event. This paper focusses on patients’ long term lived experience following a TIA.

**Methods:**

Semi structured interviews were carried out with patients who had a TIA between two and 24 months previously. Participants were asked about their TIA, the advice and management received and any changes made as a result of the TIA. Interviews were recorded and transcribed verbatim. Thematic content analysis involved scrutinising transcripts to look for links and associations within and between accounts in a process similar to the grounded theory approach of open coding. The category of transience emerged and was explored in more detail to examine the enduring consequences of TIA.

**Results:**

Thirty nine patients aged between 31 and 89 years were interviewed. Accounts detailed the long term impact of the TIA and the subsequent ‘at risk’ status, on the physical and psychosocial wellbeing of participants. Some participants sought to proactively manage the consequences of their TIA but found it difficult to obtain the information and support they needed, whereas others felt that no further action was needed to prevent future stroke.

**Conclusion:**

Current definitions conceptualise TIA as a transient event however our study suggests that some patients experienced long term consequences as a result of their TIA. These included anxiety and uncertainty in the light of their increased stroke risk. TIA patients need access to detailed, evidence based stroke prevention information from a credible source, and support to help them understand and apply the information over time, if they are to effectively self-manage the long term consequences of TIA and reduce their risk of future stroke.

## Background

Transient Ischemic Attack (TIA) is described as a transient episode of neurological dysfunction caused by focal brain, spinal cord or retinal ischaemia without acute infarction [1]. As a disease category TIA is found alongside other temporary or transitory disorders of a cerebral nature such as transient global amnesia. (ICD-10). Traditionally neurological dysfunction lasting less than 24 hours has resulted in a TIA diagnosis, with symptoms extending beyond this period resulting in a classification of stroke. Advances in brain imaging technology have resulted in greater specificity in the diagnostic process and some definitions now suggest that this time threshold is too broad and that TIA should be classified by clinical symptoms lasting less than one hour [1]. The significance of TIA as a predictor of major stroke is underlined by a robust evidence base with between 10–15 per cent of cases going on to experience ischemic stroke [2,3]. The strength of the evidence relating to TIA as a predictor of ischemic stroke has contributed to the development of policy and organisation of services in recent years. Internationally, recommendations for the management of TIA focus on the need for early diagnosis and medical management of the acute increased risk of ischaemic stroke [4-6] and in the United Kingdom primary care guidelines stress the importance of initiating secondary prevention measures as soon as the diagnosis is confirmed [6]. Whilst management of TIA focuses on the acute phase there is a growing body of evidence that some patients suffer consequences as a result of this ‘transient’ event and the knowledge of their subsequent increased stroke risk. Whilst there have been many studies focusing on the life post-stroke [7], research focusing on the lived experience of TIA is limited. Studies have implicated TIA in changes in function [8-10], reduced quality of life [11,12], elevated levels of fatigue, anxiety and depression [8,13], and other negative psycho-social outcomes [9,14,15]. However these studies did not include TIA patients below the age of 50 [9,11,15], followed patients for less than six months [8,11,15] and did not include an in depth exploration of patients’ lived experience [8,10,12-15]. This paper originates from a qualitative study that was part of a larger mixed methods study exploring secondary prevention of stroke. The long term consequences of TIA emerged strongly throughout the study and as a result of the paucity of research in this field this paper will address the long term experience of life post-TIA across a wide age range of patients.

## Methods

### Design

This qualitative study used semi structured interviews to explore patients’ experience and response to TIA and to any care they received as a result of the TIA. During the interviews participants were prompted to talk about medical, behavioural, lifestyle or other advice they received and to describe any changes that had occurred or that they had made to reduce their risk of future stroke. These interviews took a narrative approach in which participants were encouraged to tell the story of the TIA and life post TIA. The use of narratives to understand the individual experience of health, healthcare and rehabilitation has a long history [16-19]. This method focusses on the perspectives and perceptions of participants, highlighting the relative significance of different events, in order to capture the complexity and richness of their lived experience [20]. Ethical approval to proceed was granted by South Yorkshire Research Ethics committee, REC reference number 10/H1310/54.

### Selection and recruitment

The study was carried out across a central region of the UK which included urban and semi-rural populations from both deprived and affluent areas. Participants were recruited from five different sites within the National Health Service: one large acute hospital, three District General Hospitals and one community Trust. Participants were eligible to take part if they had attended TIA or stroke services for assessment following a suspected TIA between two and 24 months previously. Current UK guidelines [21] recommend that all patients with a suspected TIA are referred for, and receive, specialist assessment and investigation. The five recruitment sites each provided this service to all TIA patients living in the appropriate areas. There was some variation across the region in the way that this service was provided with some sites providing consultant led services to all TIA patients and others providing nurse led services to patients deemed to be at low risk for future stroke. However our choice of recruitment sites ensured that we were able to include patients from across the spectrum of severity of TIA. Participants were excluded if they had a stroke after their TIA and before their interview. This was to ensure that patients were in a position to reflect on their experiences of TIA and the follow up care they received in the aftermath of the event. Study information was sent to all potential participants by the lead stroke clinician in the relevant Trust. Participants were asked to contact the research team if they were willing to take part in the study. Recruitment took place in two stages. Initially patients were recruited using a purposive sampling frame aiming for a wide range of characteristics including age, gender, site providing care and risk of future stroke (n= 26). Patients were deemed to be at high or low risk of future stroke depending on their initial medical management, that is whether or not they were admitted to hospital (high risk) or seen in an outpatient clinic and then sent home (low risk). A subsequent sampling frame developed iteratively following preliminary analysis of interviews and a further thirteen participants (n= 13) were recruited to explore the experience of younger patients including those in paid employment, or with small children, at the time of the TIA. This was to further explore the meaning and consequence of the TIA for patients at an earlier stage in their lives. All participants signed consent forms prior to taking part in the study.

### Data collection

Data were collected using face to face interviews with 39 patients. All interviews took place in participants’ homes with the exception of one interview which was at the participant’s workplace. The first round of interviews took place between March and July 2011 and the second round between April and July 2012. Three interviewers (EC, JR and FC) carried out the interviews which followed a narrative format where participants were asked to describe what happened when they had their TIA and subsequent related events. An initial topic guide was developed and used as an aide memoire to ensure that participants were prompted to cover all points of interest during the interviews. Topics included the response to the TIA by the participant and others, the follow up care received and any changes made by the patient following the TIA. Data analysis took place concurrently with data collection and emerging themes informed the development of the subsequent sampling frame and topic guide. Later iterations of the topic guide included ideas about the cause and consequences of the TIA and the meaning of a TIA diagnosis. Interviews lasted between 30 and 90 minutes. All interviews were digitally recorded and transcribed verbatim.

### Data analysis

A thematic analysis was carried out. This focussed on the content rather than the structure of the narratives [20,22]. Analysis involved reading and re reading the transcripts (EC, JR and FC) to look for links and associations within each individual narrative in a systematic process similar to the grounded theory approach of open coding [23]. At this stage the analysis was documented by the writing of detailed within-case summaries to record aspects of each narrative that appeared to be significant. These within-case summaries were shared within the research team (EC,JR, FC and TR) and used as a basis for discussion about what each narrative revealed about the way that the individual had experienced and responded to their TIA. From these discussions a number of associations between the narratives emerged and were refined into overarching categories. Given the tension between the concept of transience inherent in the diagnosis of TIA, and our emerging findings about the enduring consequences of TIA, the overarching category of transience was selected for further analysis. This category was taken back to the narratives (EC,JR and FC) and explored in more detail to look at similarities and differences in the ways that it operated in each participant’s story. The themes of physical sequelae, practical consequences and psychological consequences were identified from this process.

NVivo version 9 was used to store and manage data (www.qsrinternational.com).

## Results

Approximately 400 patients were approached to take part in the study and around 50 patients contacted the research team to find out more about the study. Forty one participants were selected using the sampling criteria detailed above. Forty one interviews were carried out in total and 39 of these were transcribed. Two interviews were not transcribed or analysed because during the interview the participants’ disclosed that although they had initially been investigated for TIA they were ultimately given diagnoses other than TIA.

Eighteen women and 21 men were included in the study and ages ranged from 31 to 89 years. Seven participants were in work and two were primary carers for young or disabled offspring. The majority of participants were white British and three were British Pakistani. All interviews were in English and lasted between 30 and 90 minutes. Sixteen people had been admitted to hospital following their most recent TIA and so were considered high risk, 23 had been assessed but not admitted and so were considered low risk. Fourteen participants were aware that they had had more than one TIA. Length of time since the participant’s most recent TIA varied between two and 24 months.

Supporting quotations from the original data have been selected to illustrate the point being made. They are labelled with a participant number and the time elapsed between the TIA and the interview. Where the researcher’s contribution to a quotation is central to its meaning it has been included. Participants used a variety of terms to refer to their TIA. These included minor stroke, stroke and mini stroke and these terms appear in the quotations verbatim.

Table 1 provides an overview of participants.

**Table 1:** **Participant characteristics**

**Pseudonym**	**Age range**	**Time elapsed between TIA and interview**	**Ethnicity**
S1	80-89	6 months	White British
S2	60-69	9 months	White British
S3	60-69	7 months	White British
S4	70-79	9 months	White British
S5	70-79	2 months	White British
S6	80-89	3 months	White British
S9	80-89	12 months	White British
S10	50-59	15 months	White British
S11	80-89	11 months	White British
S12	50-59	15 months	White British
S13	70-79	5 months	White British
S14	70-79	10 months	White British
S15	70-79	17 months	White British
S16	80-89	6 months	White British
S17	70-79	18 months	White British
S18	30-39	7 months	White British
S19	40-49	14 months	White British
S20	60-69	12 months	White British
S21	80-89	12 months	White British
S22	60-69	9 months	White British
S23	60-69	8 months	White British
S24	30-39	5 months	White British
S25	60-69	24 months	British Pakistani
S26	60-69	19 months	British Pakistani
S27	50-59	24 months	Italian
BA1	30-39	5 months	White British
BC1	80-89	9 months	White British
BC2	50-59	12 months	White British
BC3	50-59	12 months	White British
R1	70-79	12 months	British Pakistani
R2	80-89	2 months	White British
R3	50-59	8 months	White British
R4	80-89	2 months	White British
R5	80-89	5 months	White British
D1	70-79	12 months	White British
D2	80-89	12 months	White British
D3	70-79	13 months	White British
D4	80-89	9 months	White British
D5	70-79	12 months	White British

### Transience

By implication a resolution of symptoms and return to function are to be expected post-TIA. Diagnostic and assessment criteria are founded upon the notion of a transient episode with a rapid return to a ‘normal state’. However our data question the fundamental premise upon which the disorder rests, with evidence that many of the people we interviewed felt they continued to experience a range of limitations of a physical, psychological and social nature up to 24 months after their TIA.

### Physical sequelae

Physical symptoms that occur during a TIA are similar to those of a stroke and may include one sided weakness affecting the face and body and disturbance of speech or vision. However many participants continued to experience physical symptoms, which originated with the TIA, at the time of their interview. These symptoms included: one sided weakness *“Me left hand side is a lot weaker because I've always been strong …it’s certain things I realise when I'm doing them I’m not [the] same, like strength and that, I’m not nearly as strong as I was”* (BC3, 12 months); fatigue *“I suffer from a lot of fatigue which I think can be quite a normal side effect of having a stroke”* (BA1, 5 months) and visual defects *“he give me this eye test and he just said its probably permanent the damage it’s done will be permanent… as time’s gone on it’s still there“* (D5, 12 months). In addition participants experienced more than one TIA with some participants describing recurrent TIAs *“I keep having them now”* (S23, 8 months).

### Practical consequences: long term financial and social implications

The practical consequences of the TIA extended beyond the acute stage. Participants were often, although not universally, told to avoid driving for a period, usually one month after the TIA. In some cases this meant that participants were unable to work during this time. *“I can’t drive for a month you know me job involves all round transport and everything, that’s all I thought about really”* (S12, 15 months). Some participants chose to reduce the number of hours in paid employment after their TIA, either to reduce work related stress or to manage fatigue or other ongoing physical symptoms. This reduction in the number of hours worked had financial implications for them. Increased insurance premiums for travel and driving placed further financial burden on participants. *“I’m thinking oh God insurance you know you want to go on holiday or something like that and you’re telling them you’ve had this TIA and your insurance goes up fifty pound”* (S12, 15 months).

Social relationships were altered in the light of the TIA. Participants were aware of increased anxiety in others, in particular spouses, other family members and work colleagues *“My boss, I think she just thought I was going to drop dead at any moment”* (BA1, 5 months). Intimate relationships were also affected with female participants facing uncertainty about the suitability of different methods of oral contraception in relation to their increased risk of stroke. *“Coming off the pill has meant that obviously therefore I had no contraception other than condoms,”* (S18, 7 months).

### Psychological consequences: permanence of uncertainty and anxiety

The diagnosis of TIA brought about a degree of uncertainty for many participants. Participants were aware that the diagnosis of TIA was made retrospectively when symptoms had resolved and that TIA may be a diagnosis of elimination made in the absence of confirmatory evidence. This led some participants to question whether they had indeed had a TIA and therefore whether the treatment and advice they had received was relevant to them. *“I, always at the back of my mind are there any more tests that can be done to me? Nobody said we’ve done all the tests”* (S2, 9 months).

The medical significance of TIA arises from the link between TIA and increased risk of early stroke [3]. Thus the ‘at risk’ status of the individual is inherent in the diagnosis of TIA. The impact of this ‘at risk’ diagnosis had repercussions for some participants who reported increased anxiety about their ongoing risk of future stroke. “*We’re nearly five months post [TIA] now and, you know, it’s there every day, thoughts about it are there every single day, you know, and it is becoming less so… Time is a great healer that old cliché but it is true and the further you get away from I, if you look at statistics, the further you get away from it the less likely you are for it to occur again aren’t you and after the first year the chance of it reoccurring diminishes so as time goes on but it is there all the time,”* (BA1, 5 months).

There was uncertainty about the extent to which changes in medication made as a result of their TIA reduced the risk of stroke. Some participants were reassured that the drug treatment they received would mitigate their risk of stroke, *“He [stroke consultant] stopped me from driving for a month on the first visit and he said ‘well keep taking the tablets and what have you and you’ll be alright like’ you know well basically that’s what it were*” (BC1, 9 months). However others remained uncertain about the effects of their medication on their risk of stroke and would have liked the reassurance of some form of ongoing surveillance to monitor the impact of medication and any lifestyle changes on their stroke risk. *“I’m thinking can you start taking a lower dosage you know, if your cholesterol’s gone down can you not try a lower dosage and see how you’ve gone with that? That’s what I’d really like to do you know, but not if it’s a danger to your health. But just you know, and then I think is six months normally that you go back for a blood test? Is that what you know normally happens or should you be checked more often or you know?”* (S14, 10 months).

In some cases participants were extremely proactive and sought to manage their uncertainty and anxiety by seeking further information about their own health, possible causes of their TIA and measures they could take to reduce their risk of future stroke. This was a frustrating experience for many who were unable to access the detailed personalised information they felt they needed to effectively manage their stroke risk. *“I think it would just [have] been nice to be able to sit and talk to somebody and tell them how scared I am, still am in a way, but it’s like for somebody to turn around and say ‘well this is how you can really prevent it happening again’ but there’s nobody to do it, there’s no doctors doing it, there’s no, GPs don’t do it”* (S19, 14 months).

### TIA can be a transient episode

It would be wrong to suggest that TIA had long term consequences for all participants in the sample. In some cases TIA was experienced as a transient episode with minimal or no future consequences because symptoms had resolved and participants were reassured that medication alone was sufficient to prevent any future recurrence. These participants were not looking for further information about their stroke risk or to change their behaviour to reduce the risk of future stroke. It was notable that in some of these cases participants had experienced, or were experiencing, other crises in their lives and the TIA and associated risk were understood in relation to these other events and deemed to be of lesser significance. For example one participant who had a long term disability herself and who also cared for her disabled son and her elderly mother felt *“It’s over and done with now. I’m back to normal you know what I mean? ….. I have enough to think about without thinking about a thing that might never come again.”* (BC2, 12 months).

## Discussion

There is a growing body of research about the lived experience of TIA and this paper makes an important and timely contribution to the debate around the definition and management of TIA. In this sample of 39 participants, aged between 31 and 89 years and who attended stroke services because of a probable TIA, many participants were still experiencing consequences of their diagnosis up to two years after their TIA.

These findings build on those of Gibson & Watkins who carried out a qualitative, grounded theory study with 16 participants from a vascular surgery clinic in one district general hospital who were up to three months post TIA [11]. They found that the TIA changed participants’ perception of their health as they became aware of their stroke risk. Similarly Kamara & Singh also found a discrepancy between the medical definition of TIA as an acute event and the participant’s explanation of TIA which related to the ability to recover fully from the TIA in the long term [24]. We have identified the long term consequences of TIA for participants in our sample which included 13 people under 65 of whom seven were still in work and two were primary carers of young or disabled children. Participants experienced physical, practical and psychological consequences related to the TIA and the implications of the ‘at risk’ status inherent in the TIA diagnosis.

There is ambiguity surrounding risk of future stroke for TIA patients because no one can be certain which patients will go on to have a stroke. Many people who have been diagnosed with TIA do not go on to have a stroke and others who have strokes never experience a TIA. There is also a lack of clarity about the mitigating effects of medication on future risk of stroke. The lack of certainty that can surround a TIA diagnosis further compounds this ambiguity and for some participants this translated into an uncertain and anxious patient experience and a demand for further health surveillance. These participants described an increased vigilance about their health post TIA along with a desire for long term surveillance and monitoring to provide them with reassurance. These findings reflect those of Salter et al. who explored risk in relation to osteoporosis screening [25]. Horlick Jones suggests that patients suffer damage to their ‘everyday health competence’ following a negative health event which they failed to anticipate [26]. Everyday health competence is the ability to review bodily sensations and to react in an appropriate manner, that is, to recognise when symptoms can be ignored and when action is needed. Some participants in our study described this fearful and slightly obsessive state in relation to reasserting control over their bodies following the TIA. They were unable to access the detailed personal information they felt they needed to monitor their health and this contributed to profound and long term disruption in their lives.

### Meaning of study and implications for clinicians

Current UK guidelines are limited to the acute management of TIA with a recommendation that patients should be followed up either in primary or secondary care at four weeks post TIA [4]. Our findings suggest that access to information and support in the longer term is needed to enable patients to self- manage the consequences of a TIA and to feel confident that they have taken adequate and appropriate action to reduce their risk of future stroke.

These findings have implications for primary care because TIA provides a valuable opportunity for secondary stroke prevention. The current focus on the acute management of TIA and the need to prevent immediate stroke [4,6,21] reflects the definition of TIA as a transient event. Our study shows that TIA was not a transient episode for many in our sample but rather there were long term consequences associated with TIA.

Information about secondary prevention of stroke is complex [27]. Patients need access to primary care services that provide appropriate and detailed explanations and support from a credible source beyond the acute stage to enable them to effectively self-manage their risk of future stroke. In this sample many participants were highly motivated to make lifestyle changes to address their risk of stroke but were unable to access the detailed personal information and support they felt they needed to do so successfully. Where patients are motivated to be proactive about reducing their stroke risk, support to negotiate individual goals with patients, for example, setting targets for blood pressure, blood cholesterol levels and weight, along with regular monitoring to review progress towards these targets, will promote effective self-management and the restoration of ‘everyday health competence’ [26].

Where patients view their TIA as transient and stroke risk as low priority, there is a need to balance the patient’s individual risk factors and need for patient education against their desire to regain their sense of wellbeing following the TIA.

Moreover, we believe that the role of primary care should not be limited to the secondary prevention of stroke. This paper demonstrates that TIA provides long term consequences of a physical, psychological and social nature. These long term consequences belie the ‘transient’ notion of this particular trauma. GPs and other primary care professionals are well placed to monitor the long term physical, psychological and social implications of TIA and to consider these whilst providing information, support and ongoing review of secondary stroke prevention measures.

### Unanswered questions and areas for future research

Our study shows that some TIA patients perceived that they were underserved and so future research is needed to examine models to optimise care for these patients. Further research is needed to determine which professionals have the most appropriate skill mix to deliver long term follow up support and information to TIA patients. For example, in the UK practice nurses may have skills of patient education, counselling and have the ability to engage with patients as individuals in a primary care setting whereas stroke nurse specialists have the stroke specific knowledge required but may not possess the skills or the resources to engage with patients and provide the appropriate long term support needed.

### Limitations of the study

Participants were individuals who had been referred to stroke services for specialist assessment and investigation because of a probable TIA. Current UK guidelines [21] recommend that all patients with a suspected TIA are referred for, and receive, specialist assessment and investigation. However our study did not capture patients who may have had a TIA but who did not seek medical attention, or who sought attention from their GP and were either not referred for specialist assessment or treatment, or who chose not to attend for this assessment, perhaps because their symptoms had resolved. Therefore this study may not represent the lived experience of TIA patients who choose not to act on their symptoms and it is conceivable that patients make this choice because they do not experience any long term consequences of their TIA.

In many cases there was no definitive diagnosis of TIA and so participants were recruited to the study where there was a working diagnosis of TIA. This means that some participants may not have had a TIA, for example some participants may have had a mild stroke. However all participants had been told that the most probable cause of their symptoms was a TIA and were offered follow up in accordance with this working hypothesis. Our study was concerned with patients’ experience of symptoms, diagnosis and care where the label of TIA had been operationalised. Therefore all patients had exposure to the phenomenon of interest and were eligible to contribute to the study regardless of the accuracy of the diagnostic label.

Participants were recruited between two and 24 months post TIA to ensure that they were in a position to reflect on their experiences of follow up services; however in some cases the time lapse since the TIA meant that participants struggled to recall events at the time of the TIA. Our study was not a search for truth about events but rather an exploration of the significance and consequence of TIA. Therefore these participants were able to contribute to our understanding about the way they responded to their TIA in the wider context of their lives.

## Conclusion

The medical understanding of TIA as a transient episode is not consistent with the lived experience of patients and this has implications for primary care Our study has shown that participants ranging from aged 31 to 89 years old experienced on-going physical, practical and psychological consequences up to two years after their diagnosis of TIA. TIA patients need access to detailed, evidence based stroke prevention information from a credible source, and support to help them understand and apply the information over time, if they are to effectively self-manage the long term consequences of TIA and reduce their risk of future stroke. Current UK guidelines for the management of TIA focus on the acute stage up to four weeks. There is a need to develop guidelines of best practice for the long term management of patients post TIA to ensure that stroke is prevented and quality of life is maintained.

## Abbreviation

TIA: Transient ischaemic attack
